# High-Sensitivity Cardiac Troponin as a Predictor of Atrial Fibrillation Detected After Stroke: Implications for Subsequent Cerebrocardiovascular Events

**DOI:** 10.3390/jcm14217542

**Published:** 2025-10-24

**Authors:** Bum Sung Kim, Jung Jin Park, Ji-Hoon Choi, Chang Hee Kwon, Sung Hea Kim, Kina Jeon, Yu-jin Chung, Hahn Young Kim, Hyun-Joong Kim

**Affiliations:** 1 Division of Cardiology, Department of Medicine, Konkuk University Medical Center, 120-1 Neungdong-ro, Gwangjin-gu, Seoul 05030, Republic of Korea; 20150056@kuh.ac.kr (B.S.K.); 20240371@kuh.ac.kr (Y.-j.C.); 2 Department of Neurology, Konkuk University Medical Center, 120-1 Neungdong-ro, Gwangjin-gu, Seoul 05030, Republic of Korea; parkjj@kuh.ac.kr (J.J.P.);

**Keywords:** atrial fibrillation detected after stroke, troponin, prognosis, Major Adverse Cerebral Cardiovascular Event

## Abstract

**Background:** Atrial fibrillation (AF) may be detected at the time of ischemic stroke or newly detected after stroke. While AF detected after stroke (AFDAS) is associated with poor outcomes compared to sinus rhythm, its prognostic implications relative to known-AF are inconsistent. High-sensitivity cardiac troponin (hs-cTn) is a biomarker of myocardial injury, but its role in predicting AFDAS in stroke patients is unclear. We aimed to evaluate hs-cTn as a predictor for AFDAS and to compare all-cause death, readmission for heart failure (HF) and readmission for stroke among patients with non-AF, AFDAS, and known-AF in the post-ischemic stroke period. **Methods:** From August 2014 to July 2017, 1506 patients with acute ischemic stroke were consecutively enrolled in a retrospective single-center registry. Out of these, 1019 patients were selected for analysis. The primary outcome was major adverse cardiac and cerebrovascular events (MACCE), a composite of all-cause death, HF-caused readmission, or stroke-caused readmission during follow-up. **Results**: Out of 1019 ischemic stroke patients, 121 (13.8%) developed AFDAS over a median of 22.5 months; 135 had known-AF and 763 were maintained sinus rhythm during follow-up. Elevated hs-TnI (≥99th percentile), age > 75, and left atrial volume index >34 mL/m^2^ independently predicted AFDAS. Both AFDAS and known-AF groups had a significantly increased risk of MACCE compared to the non-AF group (adjusted hazard ratio (HR) 1.85 and 1.76, respectively; *p* < 0.05 for both). The known-AF group also had a higher risk of all-cause mortality (adjusted HR 2.05, *p* = 0.02). The risks for MACCE and all-cause death did not differ significantly between the AFDAS and known-AF groups. **Conclusions:** An elevated hs-TnI level is independently associated with development of AFDAS and may serve as a valuable marker for stratifying the risk of future cerebrocardiovascular events following ischemic stroke.

## 1. Introduction

Atrial fibrillation (AF) is the most common sustained cardiac arrhythmia and is associated with substantial morbidity and mortality from stroke and heart failure [1,2]. Around 20% of patients with stroke have known-AF at the time of the index stroke, while 10–15% of stroke patients have a new or first diagnosis of AF detected after stroke (AFDAS) [3]. There is ongoing debate regarding whether known-AF and AFDAS should be considered separate entities with different patient characteristics and prognostic implications [4]; in particular, data comparing cerebrocardiovascular outcomes in patients with AFDAS and known-AF have been inconsistent [5,6]. However, compared to patients who remain in sinus rhythm after ischemic stroke, AFDAS is associated with a poor prognosis after stroke [7,8]. Cardiac troponin specifically indicates myocardial injury, and high-sensitive cardiac troponin (hs-cTn) assays allow the detection of previously undetectable levels of cardiac troponin in various clinical situations that do not exhibit acute cardiac injury. Elevated hs-cTn levels have been shown to be associated with the presence of subclinical structural heart disease or incident AF in general populations [9,10]. In patients with ischemic stroke, hs-cTn elevation has been observed without concomitant acute myocardial infarction and is related to an increased risk of death or cardiovascular events [11,12,13,14]. However, limited data exist regarding the association between cardiac troponin and the development of AFDAS in patients following ischemic stroke. Therefore, we aimed to determine the occurrence of AFDAS and to evaluate the predictive role of high-sensitivity cardiac troponin in patients with acute ischemic stroke. We also explored the risk of all-cause death and readmission for heart failure (HF) and readmission for stroke in patients with ischemic stroke in non-AF, AFDAS, and known-AF patient populations.

## 2. Methods

### 2.1. Study Population

**Figure 1:**
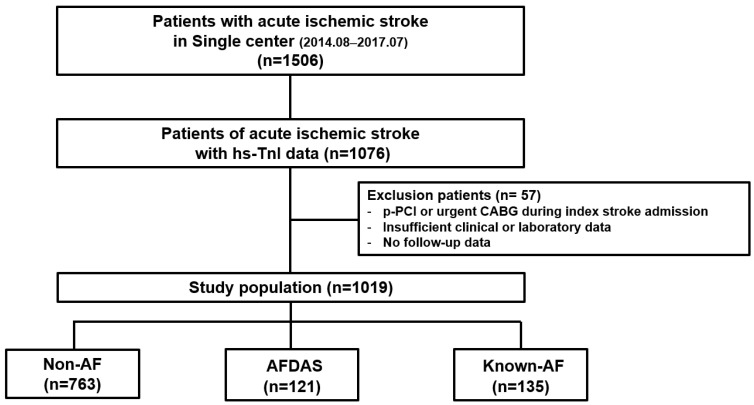
Schema of study population distribution in the registry. hs-TnI = high-sensitive cardiac troponin I; p-PCI = primary percutaneous coronary intervention; CABG = coronary artery bypass graft; AF = atrial fibrillation; AFDAS = atrial fibrillation detection after stroke.

This study is based on an observational, retrospective, single-center registry of patients with acute ischemic stroke consecutively admitted to Konkuk University Medical Center, Seoul, Korea, between August 2014 and July 2017. The electronic healthcare records of eligible patients were collected from this registry following the inclusion and exclusion criteria. Inclusion criteria were as follows: (1) patients admitted for a primary diagnosis of cerebral infarction with rapid-onset focal neurologic symptoms lasting at least 24 h, (2) 18 years of age or older, and (3) patients evaluated for high-sensitivity cardiac troponin I (hs-TnI) level at the time of admission for ischemic stroke. Exclusion criteria were (1) patients undergoing primary percutaneous coronary intervention or urgent coronary artery bypass grafting surgery during index admission with ischemic stroke, (2) patients with no follow-up visit after ischemic stroke, or (3) patients with insufficient clinical or laboratory data on initial evaluation and follow-up visit. The eligible patient inclusion process for this study is shown in Figure 1. This observational study had no influence on patient treatment because of its retrospective design, and therapies were always provided at the discretion of the attending physicians. The Institutional Review Board of Konkuk University Medical Center approved the study protocol (KUH1010848, approval date: 9 March 2017) and waived the requirement for informed consent.

### 2.2. Data Collection and High-Sensitive Cardiac Troponin I Assay

All patients underwent a complete baseline history survey, physical and neurologic examination, 12-lead electrocardiogram (ECG), and laboratory test upon admission. Clinical data, including demographic characteristics, conventional risk factors for stroke, medical co-morbidities, reperfusion therapy, and National Institutes of Health Stroke Scale (NIHSS) were collected from the registered data. Cardiac troponin I levels were assessed using the ARCHITECT STAT High-Sensitive Troponin I immunoassay on an ARCHITECT i2000SR immunoassay analyzer (Abbott Diagnostics, Lake Forest, IL, USA). The limit of detection was 1.9 ng/L. The 99th percentile upper reference limit (URL) was defined as 20.7 ng/L for men and 16.1 ng/L for women. Elevated hs-TnI level was defined as a level ≥the 99th percentile URL. Echocardiographic examinations were performed in the echocardiographic laboratory (Konkuk University Medical Center, Seoul, Republic of Korea) according to a protocol established by the American Society of Echocardiography. Clinical, laboratory, and outcome data were collected from the registry by a trained study coordinator using a standardized case report form through electrical medical record. Baseline thromboembolic risk was evaluated using the CHA2DS2-VASc score (congestive heart failure, hypertension, age ≥ 75 years [2 points], diabetes mellitus, stroke/TIA/systemic embolism [2 points], vascular disease, age 65–74 years, and sex category [female]). The prescribed medications were identified at the time of discharge from the index stroke admission. Clinical outcomes were adjudicated by two cardiologists (B.S.K, K.J). Additional information, if necessary, was obtained by further inquiry into medical records.

### 2.3. Definition of KAF and AFDAS and Study Outcomes

The presence of AF was evaluated during index ischemic stroke admission and at each follow-up visit after index admission. After discharge from index admission, patients initially underwent outpatient visitation at 1 month, and follow-up visitation was generally maintained every 3 months. A standard 12-lead ECG was checked at each visit, and for certain patients with symptoms such as palpitation, Holter monitoring was performed at the discretion of the attending physician. AF was defined by the following characteristics: (1) irregular R-R interval (when atrioventricular conduction was present), (2) absence of distinct repeating P waves, and (3) irregular atrial activity on 12-lead ECG. According to these patients’ histories of AF diagnosis, we categorized them into three groups: non-AF (patients without AF), known-AF and AFDAS. Known-AF was defined as AF before the index ischemic stroke or newly diagnosed based on the admission ECG. AFDAS was defined as newly developed AF after ischemic stroke in patients without previously documented AF during follow-up visits. Non-AF was defined as no evidence of AF either before or after index stroke. The primary outcome was major adverse cardiac and cerebrovascular event (MACCE), a composite of all-cause death, heart failure (HF)-caused readmission, or stroke-caused readmission during follow-up. Secondary outcomes were all-cause death, HF-caused readmission and stroke-caused readmission during follow-up, respectively. HF-caused readmission was defined as readmission with a primary diagnosis of HF based on major and minor clinical criteria described by the Framingham Heart Study [15]. Stroke-caused readmission was defined as readmission with a primary diagnosis of cerebral infarction with rapid-onset focal neurologic symptoms lasting at least 24 h. Reperfusion therapy was defined as intravenous tissue plasminogen activator or intra-arterial reperfusion therapy or both. Renal insufficiency was defined as an estimated glomerular filtration rate (eGFR) lower than 60 mL/min/1.73 m^2^ (using the modified diet in renal disease equation) at initial presentation.

### 2.4. Statistical Analysis

Continuous variables are presented as means with standard deviations and categorical variables as counts with percentages. To compare baseline characteristics between non-AF and other groups (AFDAS or known-AF), we used Chi-square tests for categorical variables. Continuous variables were compared using Student’s *t*-test or the Wilcoxon rank–sum test as applicable. Cumulative event rates were estimated for clinical outcomes according to type of AF by the Kaplan–Meier method and were compared using log-rank tests. Cox proportional hazard models were used to estimate hazard ratios (HR) and 95% confidence intervals (CI) for the incidence of clinical outcomes during follow-up by comparing known-AF or AFDAS groups with non-AF patients as the reference group. Multivariate Cox proportional hazard regression was performed to determine independent risk factors of AFDAS by adding significant variables (*p* < 0.10) into univariate models. Statistical analyses were performed with SPSS version 20.0 (IBM, SPSS, Chicago, IL, USA). All tests were two-tailed, and *p* < 0.05 was considered statistically significant.

## 3. Results

### 3.1. Baseline Characteristics

Among 1506 patients enrolled in the registry, 1076 patients underwent hs-TnI evaluation at the time of acute ischemic stroke admission. Of these, 57 patients were excluded based on our exclusion criteria. The patient inclusion process for this study is shown in Table 1. Finally, 1019 patients were included in the final analysis. Of these, 135 patients were assigned to the known-AF group, 121 to the AFDAS group, and 763 patients to the non-AF group. Baseline characteristics of each group are shown in Table 1. Compared with patients in the non-AF group, patients in the AFDAS or known-AF groups were older, were more likely to have had a previous myocardial infarction or HF, and had higher initial NIHSS and CHA2DS2-VASc scores. Patients with AFDAS or KAF had higher hs-TnI and lower left ventricular ejection fraction (LVEF), as well as a higher incidence of left atrium volume index (LVAI) >34 mL/m^2^ and E/e’ >15 than patients in the non-AF group. Compared with the AFDAS group, the KAF group had a higher prevalence of previous HF and stroke, higher hs-TnI level, and greater portion of patients with LVAI >34 mL/m^2^ (Supplementary Table S1).

### 3.2. Occurrence of AFDAS During Follow-Up and Independent Predictors of AFDAS

**Figure 2:**
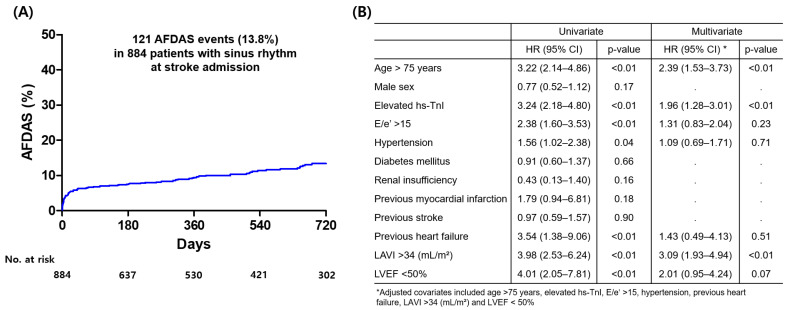
Cumulative AFDAS occurrence rate (**A**) and independent predictors of AFDAS in patients with sinus rhythm at the time of index stroke admission. (**B**) hs-TnI = high-sensitivity troponin I, AFDAS = atrial fibrillation detection after stroke; LVEF = left ventricular ejection fraction; LAVI = left atrium volume index.

Overall median follow-up period was 22.5 months (interquartile range: 5.0–38.8 month). During the follow-up period, 121 patients (13.8%) among 884 patients with sinus rhythm at the time of index stroke admission experienced AFDAS. Cumulative incidence of AFDAS is presented Figure 1. Multivariate Cox regression analysis was performed to identify predictors of AFDAS. Independent predictors of AFDAS were age >75 years, elevated hs-TnI (≥99th percentile URL of hs-TnI), and LAVI >34 mL/m^2^ (Figure 2B).

### 3.3. Clinical Outcomes According to Non-AF, AFDAS, and KAF

**Figure 3:**
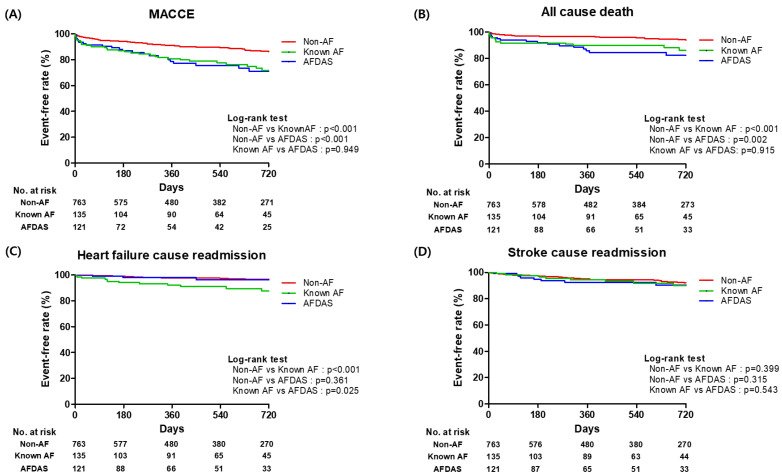
Kaplan–Meier curve of clinical outcomes according to type of AF; comparison between the non-AF group and each other groups (AFDAS or known-AF). (**A**) MACCE; (**B**) All cause death; (**C**) Heart failure caused readmission; (**D**) Stroke caused readmission. AF = atrial fibrillation; AFDAS = atrial fibrillation detection after stroke; MACCE = Major Adverse Cerebral Cardiovascular Event (a composite of all-cause death, heart failure-caused readmission or stroke-caused readmission).

Clinical outcomes of the study population, in addition to a comparison of unadjusted and adjusted HRs between the non-AF group and other two groups (AFDAS or known-AF), are presented in Table 2. The AFDAS group had a higher risk of MACCE (adjusted HR: 1.85; 95% CI: 1.23–2.79; *p* < 0.01) and tended to have a higher risk of all-cause death (adjusted HR: 1.73; 95% CI: 0.96–3.12; *p* = 0.07) compared to the non-AF group. However, there was no significant difference in the risk of heart failure-caused readmission and stroke-caused readmission between non-AF and AFDAS groups. The KAF group had a higher risk of MACCE (adjusted HR: 1.76; 95% CI: 1.17–2.64; *p* = 0.01) and all-cause death (adjusted HR: 2.05; 95% CI: 1.15–3.62; *p* = 0.02) compared to non-AF group. Comparing the non-AF group, there was a trend toward a higher risk of HF-caused readmission in known-AF group, but this finding was not statistically (adjusted HR: 1.97; 95% CI: 0.94–4.15; *p* = 0.07). There were no significant differences in risk of stroke-caused readmission between non-AF and known-AF groups. Comparing the AFDAS group with the known-AF group, there was no significant difference in clinical outcomes (Supplementary Table S2). Kaplan–Meier curves of clinical outcomes according to non-AF, AFDAS, and known-AF group designations are presented in Figure 3.

## 4. Discussion

In the present study, we investigated the predictive role of hs-cTn for AFDAS and compared subsequent clinical outcomes among non-AF, AFDAS, and known-AF groups in patients with ischemic stroke. Our main findings are as follows. First, elevated hs-TnI after ischemic stroke was a significant and independent predictor for the development of AFDAS in patients who were in sinus rhythm at the time of stroke. Second, both AFDAS and known-AF groups of patients experienced significantly higher risk of MACCE compared to patients in the non-AF group, but there was no significant difference in the risk of MACCE between the AFDAS and known-AF groups during follow-up. Third, we confirmed a substantial incidence of AFDAS (13.8%) during a median follow-up of 22.5 months.

High-sensitivity cardiac troponin, a specific and sensitive biomarker of myocardial injury, is strongly correlated with the presence of coronary artery disease and functional abnormalities such as AF, left ventricular hypertrophy, and heart failure, all of which are associated with adverse cardiovascular outcomes [16,17,18]. In real practice, patients with ischemic stroke frequently experience several cardiovascular events after stroke, such as newly detected AF and heart failure [19]. AF as a chronic disease can manifest as short, frequently subclinical paroxysms that can increase in frequency or duration over time to become sustained. This often occurs in the context of progressive atrial dysfunction or fibrosis [20]. In particular, evidence from biomarker studies suggests that the progression from sinus rhythm to AFDAS and known-AF is accompanied by a progressive increase in AF-related biomarkers, which in turn supports the concept of a continuous course of atrial myopathy in a subset of patients [21,22,23]. Our finding is that an elevated hs-TnI level on admission of ischemic stroke is an independent predictor for AFDAS. A compelling mechanistic explanation for this association is provided through an emerging conception of atrial myopathy, which posits a common underlying substrate for both AF and thromboembolic events. As recently reviewed by Papakonstantinou et al., AF and ischemic stroke may not be causally linked but rather two distinct manifestations of a primary atrial myopathy [24]. In this context, the elevated hs-TnI observed in our study may serve as a crucial biomarker for this pre-existing, subclinical atrial myopathy. Indeed, the link between structural atrial disease and myocardial biomarkers has been noted. In a substudy of the Phosphodiesterase-5 Inhibition to Improve Clinical Status and Exercise Capacity in Heart Failure with Preserved Ejection Fraction (RELAX) trial, atrial myopathy, defined by reduced atrial strain, showed an independent association with elevated troponin I levels, suggesting that subclinical myocardial injury may accompany pathological atrial remodeling [25]. Therefore, the findings of our study suggest that measurement of hs-cTn may have a predictive role for AFDAS and provide clinical evidence that it may identify patients with an underlying atrial myopathy, thereby offering a practical tool to selected high-risk patients for prolonged cardiac monitoring.

In this study, regarding for clinical outcome between non-AF and other AF groups (AFDAS or known-AF), we demonstrated that patients in other AF groups had a more unfavorable prognosis compared to those with non-AF. This finding is consistent with previous cohort studies, which have demonstrated that any form of AF is associated with a higher risk of stroke recurrence and mortality after an index stroke [7,26]. Regarding the clinical outcomes between AFDAS and known-AF, our study findings suggest that the risk of clinical outcomes is similar, which contrasts with some previous literature. Specifically, we found no significant difference in the risk of MACCE or all-cause death. This differs from some cohort studies that reported a lower mortality risk in AFDAS patients [6,27]. Regarding stroke recurrence, our findings were also comparable between the two groups, whereas a large meta-analysis suggested a lower risk for AFDAS [28]. We presumed that the discrepancy in mortality and stroke outcomes between our study and others could be attributed to a methodological factor of AF detection strategy. Our study’s definition of AFDAS, based on AF detection via 12-lead ECG during follow-up, has significant implications. This methodology likely selects patients with a more substantial AF burden than those identified through prolonged cardiac monitoring, which may explain how the differences in clinical outcomes between our AFDAS and known-AF cohorts were mitigated. This aligns with the previous findings that ECG-detected AF was linked to a higher risk of clinical events than prolonged cardiac monitor-detected AF [29].

This study had several limitations. First, the study design was nonrandomized, retrospective, and observational, which may have significantly affected the results due to confounding factors. Although multivariate analysis was performed to adjust for these potential confounding factors, unmeasured variables could not be corrected. Second, although this registry consecutively included patients with ischemic stroke, hs-TnI levels were not evaluated for all patients in the registry. Patients without initial hs-TnI data were excluded from the analysis, which may have introduced selection bias. Third, restoration of sinus rhythm and use of anticoagulant during follow-up might be an effective treatment option for patients with AFDAS to reduce the burden of cardiovascular events. Unfortunately, we did not have information concerning the rhythm status and use of anticoagulants of the AFDAS population from follow-up visits. Fourth, during index stroke admission, the monitoring protocol for AFDAS assessment was not uniform across the cohort and our assessment of AFDAS during follow-up visits was based on a 12-lead ECG, which is a recognized limitation. Current literature and guidelines agree that longer cardiac monitoring increases the likelihood of AF detection [2,30]. Nonetheless, while our approach is potentially less sensitive than continuous monitoring, it reflects real-world clinical practice and the daily management of post-ischemic stroke patients.

## 5. Conclusions

The elevated hs-TnI after ischemic stroke was an independent predictor for the development of AFDAS in the post-ischemic stroke period. Clinical outcomes for the AFDAS group were comparable to those of the KAF group but were associated with more unfavorable outcomes than those of the non-AF group. Our findings support the growing body of evidence suggesting that hs-TnI may aid in individualized risk stratification of future cerebrocardiovascular events.

## Figures and Tables

**Table 1:** Baseline characteristics of the study population according to type of atrial fibrillation.

Overall Population	Non-AF	AFDAS	*p*-Value (Non-AF vs. AFDAS)	Known-AF	*p*-Value (Non-AF vs. KAF)
(*n* = 1019)	(*n* = 763)	(*n* = 121)		(*n* = 135)	
Age (years)	69.2 ± 13.7	77.2 ± 10.7	<0.01	77.5 ± 8.9	<0.01
Age > 75 years old	308 (40.4)	83 (68.6)	<0.01	95 (70.4)	<0.01
Male sex	432 (56.6)	60 (49.6)	0.15	73 (45.1)	0.58
Medical history					
Hypertension	474 (62.1)	87 (71.9)	0.04	98 (72.6)	0.02
Diabetes mellitus	235 (30.8)	36 (29.8)	0.82	50 (37.0)	0.15
Current smoking	182 (23.9)	15 (12.4)	<0.01	12 (8.9)	<0.01
Renal insufficiency	79 (10.4)	8 (6.6)	0.19	19 (14.1)	0.21
Dyslipidemia	154 (20.2)	14 (11.6)	0.03	29 (21.5)	0.73
Previous myocardial infarction	18 (2.4)	10 (8.3)	<0.01	10 (7.4)	<0.01
Previous PCI	29 (3.8)	7 (5.8)	0.31	11 (8.1)	0.02
Previous CABG	13 (1.7)	4 (3.3)	0.23	3 (2.2)	0.67
Previous heart failure	13 (1.7)	7 (5.8)	<0.01	26 (19.3)	<0.01
Previous stroke	134 (17.6)	23 (19.0)	0.69	41 (30.4)	<0.01
Echocardiographic parameters					
LVEF (%)	66.9 ± 8.3	63.2 ± 12.4	<0.01	60.5 ± 12.5	<0.01
LVEF < 50%	26 (3.4)	15 (12.4)	<0.01	20 (14.8)	<0.01
LAVI > 34 (mL/m^2^)	356 (46.7)	94 (77.7)	<0.01	124 (91.9)	<0.01
E/e’ >15	80 (10.5)	31 (25.6)	<0.01	36 (26.7)	<0.01
Laboratory parameters					
hs-TnI (ng/L)	49.06 ± 30.07	56.50 ± 32.04	0.03	140.86 ± 105.87	<0.01
>99 percentile hs-TnI	188 (24.6)	53 (43.8)	<0.01	70 (51.9)	<0.01
Hemoglobin (g/dL)	13.6 ± 2.0	13.5 ± 2.0	0.45	13.1 ± 1.8	0.01
Creatinine (mg/dL)	1.1 ± 0.58	1.0 ± 0.89	0.39	1.1 ± 0.46	0.69
LDL cholesterol (mg/dL)	103.3 ± 44.1	98.3 ± 29.8	0.23	94.1 ± 33.3	0.04
Total cholesterol (mg/dL)	174.6 ± 48.4	164.9 ± 36.6	0.03	154.1 ± 40.9	<0.01
Initial NIHSS	4.3 ± 3.3	8.1 ± 5.3	<0.01	8.5 ± 4.5	<0.01
CHA2DS2-VASc Score	4.4 ± 1.4	5.3 ± 1.3	<0.01	5.6 ± 1.4	<0.01
Reperfusion therapy					
Intravenous thrombolysis	71 (9.3)	27 (22.3)	<0.01	34 (25.2)	<0.01
Endovascular treatment	33 (4.3)	11 (9.1)	0.03	24 (17.8)	<0.01
Medications					
Aspirin	579 (75.9)	46 (38.0)	<0.01	27 (20.0)	<0.01
Clopidogrel	329 (43.1)	28 (23.1)	<0.01	22 (16.3)	<0.01
Warfarin	12 (1.6)	19 (15.7)	<0.01	35 (25.9)	<0.01
DOAC	9 (1.2)	37 (30.6)	<0.01	60 (44.4)	<0.01
Statin	663 (86.9)	99 (81.8)	0.13	105 (77.8)	<0.01

Values are mean ± standard deviation or *n* (%). AF = atrial fibrillation; AFDAS = atrial fibrillation detection after stroke; KAF = known atrial fibrillation; PCI = percutaneous coronary intervention; CABG = coronary artery bypass graft; LVEF = left ventricular ejection fraction; LAVI = left atrium volume index; hs-TnI = high-sensitive cardiac troponin I; LDL = low density lipoprotein cholesterol; NIHSS = National Institutes of Health Stroke Scale; DOAC= direct oral anticoagulant.

**Table 2:** Comparison of risks of clinical outcomes between the non-AF group and the AFDAS or known-AF groups.

	Non-AF	AFDAS	Known-AF
	(*n* = 763)	(*n* = 121)	(*n* = 135)
MACCE	116 (15.2)	33 (27.3)	38 (28.1)
Unadjusted HR (95% CI)	1 [Reference]	2.21 (1.52–3.20)	2.28 (1.57–3.32)
*p*-value		<0.01	<0.01
Adjusted HR (95% CI) *	1 [Reference]	1.85 (1.23–2.79)	1.76 (1.17–2.64)
*p*-value		<0.01	0.01
All-cause death	49 (6.4)	19 (15.7)	19 (14.1)
Unadjusted HR (95% CI)	1 [Reference]	2.32 (1.35–3.99)	2.77(1.61–4.78)
*p*-value		<0.01	<0.01
Adjusted HR (95% CI) *	1 [Reference]	1.73 (0.96–3.12)	2.05 (1.15–3.62)
*p*-value		0.07	0.02
Heart failure-caused readmission	25 (3.3)	6 (5.0)	14 (10.4)
Unadjusted HR (95% CI)	1 [Reference]	1.51 (0.62–3.71)	3.69 (1.89–7.20)
*p*-value		0.36	<0.01
Adjusted HR (95% CI) *	1 [Reference]	0.89 (0.34–2.38)	1.97 (0.94–4.15)
*p*-value		0.83	0.07
Stroke-caused readmission	58 (7.6)	14 (11.6)	11 (8.1)
Unadjusted HR (95% CI)	1 [Reference]	1.36 (0.74–2.50)	1.32 (0.68–2.54)
*p*-value		0.31	0.41
Adjusted HR (95% CI) *	1 [Reference]	1.39 (0.74–2.62)	1.51 (0.76–2.99)
*p*-value		0.29	0.23

AF = atrial fibrillation; HR = hazard ratio; CI = confidence interval; MACCE = Major Adverse Cerebral Cardiovascular Event (a composite of all-cause death, heart failure-caused readmission or stroke-caused readmission). * Adjusted covariates included age >75 years, LVEF < 50%, previous heart failure, and previous stroke.

## Data Availability

The datasets used during this research are not publicly available because of privacy and ethical restrictions. However, they are available from the corresponding author on reasonable request.

## Supplementary Materials

The following supporting information can be downloaded at: https://www.mdpi.com/article/10.3390/jcm14217542/s1, Table S1: Baseline characteristics between the atrial fibrillation detected after stroke (AFDAS) and known atrial fibrillation group; Table S2: Comparison of risks of clinical outcomes between the atrial fibrillation detected after stroke (AFDAS) and known atrial fibrillation group.
