# Supplementary material for: High-Sensitivity Cardiac Troponin as a Predictor of Atrial Fibrillation Detected After Stroke: Implications for Subsequent Cerebrocardiovascular Events
Source: J Clin Med. 2025 Oct 24;14(21):7542. doi: 10.3390/jcm14217542 (PMC12608220; doi:10.3390/jcm14217542)
Supplement: Supplementary file 1 [file jcm-14-07542-s001.zip › jcm-3930644-supplementary.pdf]

Supplement S1. Baseline characteristics between the atrial fibrillation detected after stroke (AFDAS) and known atrial fibrillation group.

	AFDAS (n=121)	Known-AF (n=135)	*P*-value
Age (years)	77.2 ± 10.7	77.5 ± 8.9	0.79
Age > 75 years old	83 (68.6)	95 (70.4)	0.76
Male sex	60 (49.6)	73 (45.1)	0.47
**Medical history**			
Hypertension	87 (71.9)	98 (72.6)	0.91
Diabetes mellitus	36 (29.8)	50 (37.0)	0.22
Current smoking	15 (12.4)	12 (8.9)	0.36
Renal insufficiency	8 (6.6)	19 (14.1)	0.05
Dyslipidemia	14 (11.6)	29 (21.5)	0.03
Previous myocardial infarction	10 (8.3)	10 (7.4)	0.79
Previous PCI	7 (5.8)	11 (8.1)	0.46
Previous CABG	4 (3.3)	3 (2.2)	0.59
Previous heart failure	7 (5.8)	26 (19.3)	<0.01
Previous stroke	23 (19.0)	41 (30.4)	0.04
**Echocardiographic parameters**			
LVEF (%)	63.2 ± 12.4	60.5 ± 12.5	0.09
LVEF < 50%	15 (12.4)	20 (14.8)	0.57
LAVI > 34 (mL/m^2)	94 (77.7)	124 (91.9)	<0.01
E/e' >15	31 (25.6)	36 (26.7)	0.85
**Laboratory parameters**			
hs-TnI (ng/L)	56.50 ± 32.04	140.86 ± 105.87	0.04
> 99perceintil hs-TnI	53 (43.8)	70 (51.9)	0.19
Hemoglobin (g/dL)	13.5 ± 2.0	13.1 ± 1.8	0.17
Creatinine (mg/dL)	1.0 ± 0.89	1.1 ± 0.46	0.47
LDL cholesterol (mg/dL)	98.3 ± 29.8	94.1 ± 33.3	0.54
Total cholesterol (mg/dL)	164.9 ± 36.6	154.1 ± 40.9	0.03
**Initial NIHSS**	8.1 ± 5.3	8.5 ± 4.5	0.49
**CHA2DS2-VASc Score**	5.3 ± 1.3	5.6 ± 1.4	0.19
**Reperfusion therapy**			
Intravenous thrombolysis	27 (22.3)	34 (25.2)	0.59
Endovascular treatment	11 (9.1)	24 (17.8)	0.04
**Medications**			
Aspirin	46 (38.0)	27 (20.0)	<0.01
Clopidogrel	28 (23.1)	22 (16.3)	0.17
Warfarin	19 (15.7)	35 (25.9)	0.05
DOAC	37 (30.6)	60 (44.4)	0.02

Statin	99 (81.8)	105 (77.8)	0.42

Values are mean ± standard deviation or n (%)

AF = atrial fibrillation; AFDAS = atrial fibrillation detection after stroke; PCI = percutaneous coronary intervention; CABG = coronary artery bypass graft; LVEF = left ventricular ejection fraction; LAVI = left atrium volume index; hs-cTnI = high-sensitive cardiac troponin I; LDL = low density lipoprotein cholesterol; NIHSS = National Institutes of Health stroke scale; DOAC= direct oral anticoagulant

Supplement 2. Comparison of risks of clinical outcomes between the atrial fibrillation detected after stroke (AFDAS) and known atrial fibrillation group.

	AFDAS (n=121)	Known-AF (n=135)	Unadjusted HR (95% CI)	*P*-value	Adjusted HR* (95% CI)	*P*-value
MACCE	33 (27.3)	38 (28.1)	1.01 (0.62-1.63)	0.99	0.96 (0.58-1.59)	0.89
All-cause death	19 (15.7)	19 (14.1)	0.97 (0.50-1.85)	0.91	0.90 (0.46-1.76)	0.76
Heart failure cause readmission	6 (5.0)	14 (10.4)	3.34 (1.09-10.25)	0.04	3.18 (0.99-10.20)	0.05
Stroke cause readmission	14 (11.6)	11 (8.1)	0.77 (0.34-1.75)	0.54	0.75 (0.32-1.73)	0.49

AF = atrial fibrillation; HR = hazard ratio; CI = confidence interval; MACCE = Major Adverse Cerebral Cardiovascular Event (a composite of all-cause death, heart failure cause readmission or stroke cause readmission)

*Adjusted covariates included age >75 years, LVEF < 50%, previous heart failure and previous stroke
